# A Gene Variation at the ZPR1 Locus (rs964184) Interacts With the Type of Diet to Modulate Postprandial Triglycerides in Patients With Coronary Artery Disease: From the Coronary Diet Intervention With Olive Oil and Cardiovascular Prevention Study

**DOI:** 10.3389/fnut.2022.885256

**Published:** 2022-06-17

**Authors:** Juan F. Alcala-Diaz, Antonio P. Arenas-de Larriva, Jose D. Torres-Peña, Fernando Rodriguez-Cantalejo, Oriol A. Rangel-Zuñiga, Elena M. Yubero-Serrano, Francisco M. Gutierrez-Mariscal, Magdalena P. Cardelo, Raul M. Luque, Jose M. Ordovas, Pablo Perez-Martinez, Javier Delgado-Lista, Jose Lopez-Miranda

**Affiliations:** ^1^Lipids and Atherosclerosis Unit, Department of Internal Medicine, Maimonides Biomedical Research Institute of Córdoba (IMIBIC), Reina Sofía University Hospital, University of Córdoba, Córdoba, Spain; ^2^Centro de Investigación Biomédica en Red de Fisiología de la Obesidad y Nutricion (CIBERobn), Instituto de Salud Carlos III, Madrid, Spain; ^3^Biochemical Laboratory, Reina Sofía University Hospital, Córdoba, Spain; ^4^Department of Cell Biology, Physiology, and Immunology, Maimonides Biomedical Research Institute of Córdoba (IMIBIC), Reina Sofía University Hospital, University of Córdoba, Córdoba, Spain; ^5^Jean Mayer USDA Human Nutrition Research Center on Aging, Tufts University, Boston, MA, United States; ^6^Instituto Madrileño de Estudios Avanzados en Alimentación (IMDEA-Food), Madrid, Spain; ^7^International Advisory Board, University Camilo José Cela, Madrid, Spain

**Keywords:** postprandial triglycerides, diet, coronary artery disease, single nucleotide polymorphism, SNP, nutrigenomics, nutrigenetics

## Abstract

**Background and Aims:**

rs964184 variant in the ZPR1 gene has been associated with blood lipids levels both in fasting and postprandial state and with the risk of myocardial infarction in high-risk cardiovascular patients. However, whether this association is modulated by diet has not been studied.

**Objective:**

To investigate whether the type of diet (low-fat or Mediterranean diets) interacts with genetic variability at this loci to modulate fasting and postprandial lipids in coronary patients.

**Materials and Methods:**

The genotype of the rs964184 polymorphism was determined in the Cordioprev Study population (NCT00924937). Fasting and Postprandial triglycerides were assessed before and after 3 years of dietary intervention with either a Mediterranean or a low-fat diet. Postprandial lipid assessment was done by a 4-h oral fat tolerance test (OFTT). Differences in triglycerides levels were identified using repeated-measures ANCOVA.

**Results:**

From 523 patients (85% males, mean age 59 years) that completed the OFTT at baseline and after 3 years of intervention and had complete genotype information, 125 of them were carriers of the risk allele G. At the start of the study, these patients showed a higher fasting and postprandial triglycerides (TG) plasma levels. After 3 years of dietary intervention, G-carriers following a Mediterranean Diet maintained higher fasting and postprandial triglycerides, while those on the low-fat diet reduced their postprandial triglycerides to similar values to the population without the G-allele.

**Conclusion:**

After 3 years of dietary intervention, the altered postprandial triglyceride response induced by genetic variability in the rs964184 polymorphism of the ZPR1 gene can be modulated by a low-fat diet, better than by a Mediterranean diet, in patients with coronary artery disease.

## Introduction

The study of the effect of genetic differences between individuals on their response to a specific nutrient or dietary pattern for a specific health outcome (Nutrigenetics), and the study of the effect of foods on gene expression (Nutrigenomics) have enabled the development of the field of personalized nutrition and precision healthcare in the last years (1). Personalized nutritional counseling based on a person’s genetic background may improve the outcomes of a specific dietary intervention and provide a novel therapeutic strategy for better control of cardiovascular disease or its related risk factors, such as diabetes, obesity, or blood lipid levels (2–4).

Recently, there has been a growing recognition of the role of postprandial lipemia and the metabolism of triglycerides-rich proteins in the development and progression of atherosclerosis (5). Thus, postprandial triglyceride levels independently predict the risk of coronary artery disease (CAD), peripheral vascular disease, and cerebrovascular disease and may be stronger predictors of cardiovascular disease (CVD) than fasting triglycerides (6–10). This may be due to the proinflammatory, procoagulant, and prooxidant environment that characterizes the physiological response to dietary intakes, particularly a high-fat meal (11, 12). Specifically, the main mechanisms proposed for atherosclerosis induced by postprandial lipemia include: (1) endothelial dysfunction induced by increased plasma TG with decreased serum nitrite/nitrate levels and flow-mediated dilation, (2) an increase in reactive oxygen species mediated through mitochondrial dissociation and beta-oxidation, (3) increased expression of adhesion factors at the subendothelial level, (4) activation of complement component factor 3, and (5) upregulation of proinflammatory and pro-apoptotic genes in endothelial cells following fat intake (12–15).

Several factors have been described as influencing postprandial lipemia (PPL) response. These include, among others, diet, age, gender and genetics (16–20). Previous studies have evaluated the impact of various nutrients on PPL, indicating that carbohydrate quality, dietary fat content, and polyphenols may be key modulators (21). Among dietary patterns, the Mediterranean diet (MedDiet), with a high proportion of fiber-rich foods, whole grain cereals, vegetables, fruit, polyphenol-rich foods, and a low intake of saturated fatty acids and a high proportion of MUFA, has been proposed as a very attractive option for the prevention and treatment of cardiometabolic risk factors with positive effects on PPL (21). However, very few clinical trials have addressed the long-term effect of other dietary patterns on PPL. Due that the postprandial increase in plasma lipids is related to the amount of ingested fats, reducing fat consumption through a low-fat (LF) diet pattern could be an alternative option that remains to be fully elucidated (22, 23).

Regarding genetics, different common and rare gene variants have been related to plasma triglycerides (TG) (20). Interestingly, a single nucleotide polymorphism (SNP) located in the ZPR1 gene (rs964184) has been identified through genome-wide association study (GWAS) to be associated with the magnitude of postprandial TG response, with G-allele carriers of this SNP at greater risk of high plasma TG values during postprandial state compared to the reference allele carriers (24). The ZPR1 gene, located near the apolipoprotein gene cluster APOA1/C3/A4/A5 on chromosome 11, codes a regulatory protein that binds various transcription factors and interacts with the triglyceride-associated gene APOA5 (25). Its genetic variant rs964184 has been related to fasting TG (26), metabolic syndrome (27, 28), type 2 diabetes mellitus (29, 30), non-alcoholic fatty liver disease (NAFLD) (31), lipid response to fenofibrate (32), subclinical atherosclerosis (33), and coronary artery disease (34, 35). Although this variant has also been involved in plasma fasting lipids’ response to dietary fat (36), to date, there is no information on how different dietary patterns can influence the postprandial lipid response in relation to this SNP.

In our group, we have reported in the population of the CORDIOPREV study (ClinicalTrials.gov Identifier: NCT00924937) how the long-term consumption of a MedDiet may modulate PPL mainly in patients with type 2 diabetes mellitus (37), and the existence of a gene-diet interaction between genetic variants of ApoE and MedDiet (38). The current study aimed to investigate whether long−term consumption of two healthy dietary patterns (LF diet or MedDiet) interacts with rs964184 SNP at the ZPR1 gene to modulate postprandial lipemia in coronary heart disease patients.

## Materials and Methods

### Study Design and Subjects

The current study was conducted based on the Coronary Diet Intervention With Olive Oil and Cardiovascular Prevention (CORDIOPREV) study, which is a prospective, randomized, controlled trial including 1002 patients with coronary heart disease (CHD) to compare the effects of the consumption of two different dietary patterns (LF diet versus MedDiet) on the incidence of cardiovascular events of patients with coronary disease after 7 years of intervention (ClinicalTrials.gov Identifier: NCT00924937). The design, rationale and baseline characteristics of the CORDIOPREV study have been described elsewhere (39). Inclusion and exclusion criteria are shown in Supplementary Table 1. Briefly, patients were eligible if they were older than 20 but younger than 76 years old, had established CHD, were thought to follow a long-term dietary intervention and had no severe diseases or an expected life expectancy lower than the length of the study. No medication was defined as an exclusion criteria. Details of the dietary intervention have been published previously (40). In summary, the dietary models were as follows: (1) MedDiet, with a minimum of 35% of calories as fat (22% MUFA, 6% PUFA, and <10% saturated), 15% protein, and a maximum of 50% carbohydrates, and (2) LF diet, with high complex carbohydrates, <30% of total fat (<10% saturated fat, 12–14% MUFA and 6–8% PUFA), 15% protein, an a minimum of 55% carbohydrates. The 14-point Mediterranean Diet Adherence Screener (MEDAS) and a 9-point Low-Fat diet adherence were used to assess dietary adherence (41). There were no energy restrictions in place.

From 1002 patients included in the CORDIOPREV study, a total of 557 patients completed an oral fat test tolerance (OFTT) at baseline and after 3 years of intervention and were selected for genotyping.

The Ethics Committee of Reina Sofía University Hospital approved the trial protocol (n°1496/27/03/2009), which follows the Helsinki declaration and the charter of good clinical practices. The experimental protocol conforms to international ethical standards, and written informed consent was obtained from all the subjects.

### Oral Fat Tolerance Tests

At the start of the CORDIOPREV Study and after 3 years of dietary intervention, patients received an OFTT using a weight-adjusted meal (0.7 g fat and 5 mg cholesterol per kg body weight) with 12% saturated fatty acids (SFA), 10% polyunsaturated fatty acids (PUFA), 43% monounsaturated fatty acids (MUFA), 10% protein, and 25% carbohydrates (CHO). The methodology followed for the OFTT has been described in the previous work of our group (42).

The patients fasted before beginning the test (no food or medicines) for 12 h. They were advised to refrain from smoking throughout the fasting period and from consuming alcohol for the previous 7 days. They were also instructed not to engage in any strenuous physical activity the day before the exam.

At 08:00 a.m., the patients arrived at the clinical center and a fasting blood sample and anthropometric measures were taken. Then, they consumed a fat-rich meal challenge under observation during a maximum period of 20 min. The participants were allowed to drink water but were not allowed to eat other foods or drink other beverages during the test period. Blood samples for postprandial biochemical measurements were taken every hour for the following 4 h.

### Laboratory Tests and Genotyping

Biochemical measurements and DNA isolation have been previously described (43). 200 uL of DNA (50 ng/uL) isolated from blood samples were sent to the Human Genomics Facility of the Genetic Laboratory of the Department of Internal Medicine at Erasmus MC (Rotterdam, Netherlands), where genotyping was performed using the Illumina GSA beadchip GSA MD v1 (Illumina GSA Arrays “Infinium iSelect 24 × 1 HTS Custom Beadchip Kit”). In this research, we analyzed the rs964184 variation previously reported in the literature linked with lipid metabolism and coronary artery disease (24, 34).

### Statistical Analysis

All variables had their distributions checked for normality, and skewed variables were normalized using log10 as needed. Depending on the presence of two or more groups in each comparison, continuous variables were compared using Student’s “*t*” and analysis of variance (ANCOVA). To quantify the magnitude of change during the postprandial state, we evaluated total (AUC) and incremental (iAUC) area under the curves of the different postprandial parameters using the trapezoid method, as in earlier works by our group (42). The genotype distributions did not differ from those predicted by Hardy-Weinberg (*P* > 0.05). Due to the low frequencies of homozygous patients for the minor allele (G/G = 7), patients carrying some risk allele (G/G or C/G) were grouped for analysis. Differences in postprandial values were identified using repeated-measures ANOVA, adjusting by age, gender, lipid-lowering drugs, and body mass index (BMI). We calculated the overall gene influence (SNP *p*-value), the kinetics of the response (Time *p*-value) and the interaction of both factors (Time × SNP *p*-value). An SNP-diet interaction term was used to evaluate gene-diet interactions. Bonferroni’s correction was used for multiple comparisons when necessary. Differences were considered to be significant when *p* < 0.05. All analyses were performed using R software (version 3.6.1).

## Results

**FIGURE 1:**
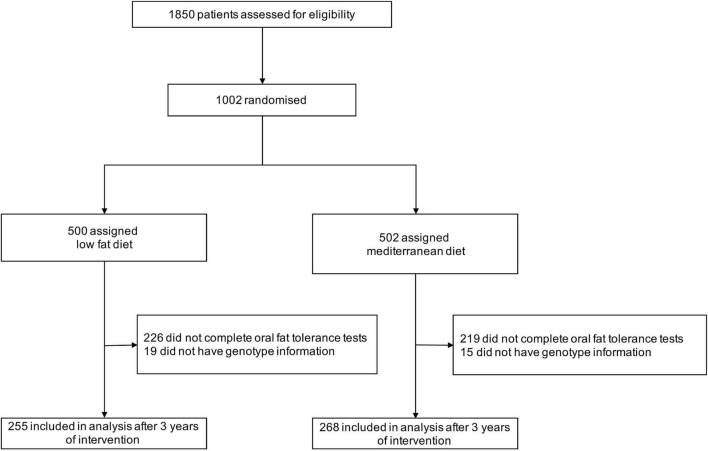
Flow-chart of the study.

From 1002 patients included in the CORDIOPREV study, a total of 557 patients completed OFTT at baseline and after 3 years of intervention. Of those, 523 had genotype information and were included in the present analysis (268 in the MedDiet group and 255 in the LF diet group). A flowchart of the participant inclusion is shown in Figure 1.

Baseline characteristics according to genotype rs964184 SNP are shown in Table 1. There were no significant genotype-related differences for the variables examined, except for the higher HDL-cholesterol (HDL-c) and lower fasting triglycerides observed in C/C subjects compared with G-allele carriers (*p* < 0.01). Baseline and demographic data according to the dietary pattern received are shown in Supplementary Table 2. Changes in adherence to the MedDiet and LF, measured by the scoring scales used in the study (MEDAS and 9-point low-fat adherence) during the follow-up are shown in Supplementary Figure 1.

**TABLE 1:** Baseline characteristics by genotype according to ZPR1 SNP rs964184.

	rs964184		
	C/C	C/G + G/G	*p*-value
*N* (%)	398 (76.1)	125 (23.9)	
Male *n* (%)	334 (83.9)	111 (88.8)	0.11
Age (years)	59.3 ± 0.4	58.1 ± 0.8	0.18
T2DM *n* (%)	172 (43.2)	58 (46.4)	0.54
Weight (kg)	84.4 ± 0.7	85.4 ± 1.4	0.51
WC (cm)	103.9 ± 0.6	104.5 ± 1.1	0.60
BMI (kg/m^2^)	30.7 ± 0.2	31.1 ± 0.4	0.41
TC (mg/dL)	159.2 ± 1.5	162.1 ± 3.1	0.37
LDL-c (mg/dL)	89.4 ± 1.3	89.5 ± 2.3	0.98
HDL-c (mg/dL)	43.2 ± 0.5	39.9 ± 0.8	0.002*
TG (mg/dL)	123.7 ± 3.9	154.3 ± 9.5	<0.001*
ApoA1 (mg/dL)	126.3 ± 1.1	123 ± 1.8	0.13
ApoB (mg/dL)	68.8 ± 0.8	71.4 ± 1.6	0.13
Lp(a) (mg/dL)	39.7 ± 2.1	41.1 ± 4	0.76
hs-CRP (mg/L)	2.9 ± 0.2	3.1 ± 0.3	0.52
Physical activity (MET-min/day)	271 ± 12.5	280 ± 21.5	0.720
Former smoker *n* (%)	260 (65.3)	85 (68)	0.231
Current smoker *n* (%)	34 (8.5)	15 (12)	0.231

*Values are expressed as frequencies (%) or mean ± SEM.*

*Continuous variables have been calculated using the ANOVA test.*

*Qualitative variables were compared using Fisher’s exact test.*

*ApoA1, apolipoprotein A1; ApoB, apolipoprotein B; BMI, body mass index; cm, centimeters; HDL-c, high-density lipoprotein cholesterol; hs-CRP, high sensitivity C-reactive protein; kg, kilograms; kg/m^2^, kilograms per square meter; LDL-c, low-density lipoprotein cholesterol; Lp(a), lipoprotein a; MET, metabolic equivalent of task; mg/dL, milligrams per deciliter; mg/L, milligrams per liter; T2DM, type 2 diabetes mellitus; TC, total cholesterol; TG, triglycerides; WC, waist circumference.*

*P < 0.05.

Table 2 shows the fasting lipid profile and body mass index (BMI) before and after the dietary intervention. No significant changes were observed in BMI, total cholesterol (TC), LDL-c, HDL-c, triglycerides, ApoA1, ApoB or Lp(a) after intervention adjusting by potential confounders (gender, age, BMI, lipid-lowering drugs). After 3 years of intervention, no gene-diet interactions were identified in fasting lipid changes. There were also no differences in physical activity between genotypes after the intervention (*p* = 0.982).

**TABLE 2:** Fasting lipid profile, body mass index at baseline, and their changes after 3 years of intervention according to rs964184 SNP and dietary patterns.

Baseline					

	LF diet		MedDiet		
	C/C (*n* = 188)	C/G + G/G (*n* = 67)	C/C (*n* = 210)	C/G + G/G (*n* = 58)	*P*_diet × genotype_
BMI (kg/m^2^)	30.6 ± 0	31.7 ± 1	30.8 ± 0	30.3 ± 1	0.653
TC (mg/dL)	157 ± 2	163 ± 4	161 ± 2	161 ± 5	0.197
LDL-c (mg/dL)	88 ± 2	90 ± 3	91 ± 2	89 ± 4	0.141
HDL-c (mg/dL)	43 ± 1^a^	38 ± 1^a^	43 ± 1	42 ± 1	0.267
TG (mg/dL)	126 ± 5^b^	168 ± 9^b^	127 ± 5^c^	140 ± 8^c^	0.130
ApoA1 (mg/dL)	130 ± 1	123 ± 2	130 ± 2	132 ± 3	0.091
ApoB (mg/dL)	71 ± 1	76 ± 2	73 ± 1	76 ± 3	0.332
Lp(a) (mg/dL)	41 ± 3	40 ± 5	38 ± 3	42 ± 6	0.508
**After 3 years of intervention**					
BMI (kg/m^2^)	30 ± 0	31 ± 1	31 ± 0	30 ± 1	0.674
TC (mg/dL)	157 ± 2	162 ± 4	161 ± 2	168 ± 4	0.926
LDL-c (mg/dL)	92 ± 2	97 ± 4	95 ± 2	95 ± 3	0.289
HDL-c (mg/dL)	41 ± 1^d^	37 ± 1^d^	41 ± 1	40 ± 1	0.546
TG (mg/dL)	119 ± 4^e^	154 ± 17^e^	120 ± 5^f^	176 ± 20^f^	0.087
ApoA1 (mg/dL)	128 ± 2	121 ± 3	129 ± 2	129 ± 3	0.221
ApoB (mg/dL)	74 ± 2	79 ± 3	79 ± 3	80 ± 3	0.645
Lp(a) (mg/dL)	51 ± 4	49 ± 6	45 ± 4	50 ± 8	0.987

*Values expressed as mean ± SEM.*

*P-values were calculated using the ANOVA test adjusted by age, gender, BMI, statins, and fibrates.*

*ApoA, apolipoprotein A; ApoB, apolipoprotein B; BMI, body mass index; HDL-c, high-density lipoprotein cholesterol; LDL-c, low-density lipoprotein cholesterol; LF, low-fat; Lp(a), lipoprotein a; TC, total cholesterol; TG, triglycerides. Equal superscript letters indicate statistical differences with p-values < 0.05 between genotypes.*

**FIGURE 2:**
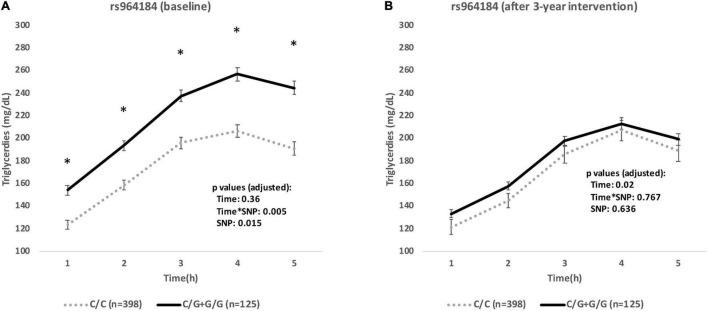
Postprandial triglycerides during baseline oral fat tolerance test **(A)** and after 3 years **(B)** of intervention by rs964184 genotypes. Values are means ± SEM. *P*-values were adjusted for age, gender, lipid-lowering drugs, and body mass index. *P < 0.05.

For the whole population, baseline postprandial triglycerides during the OFTT were higher in G-carriers at all time points (*p* < 0.05). Also a significant interaction between time × SNP was found (*p*-value = 0.005) (Figure 2A). Conversely, after 3 years of intervention, there were no differences in postprandial TG during OFTT between genotypes (*p*-value for SNP = 0.636, *p*-value for interaction time × SNP = 0.767) (Figure 2B). Changes (3 years–baseline) in postprandial TG AUC and iAUC adjusted by univariate analysis are shown in Supplementary Table 3. We identified a gene-diet interaction for the differences in postprandial TG AUC and iAUC (*p*-values SNP × diet = 0.006 and 0.031, respectively) (Supplementary Table 3).

### Low-Fat Intervention

**FIGURE 3:**
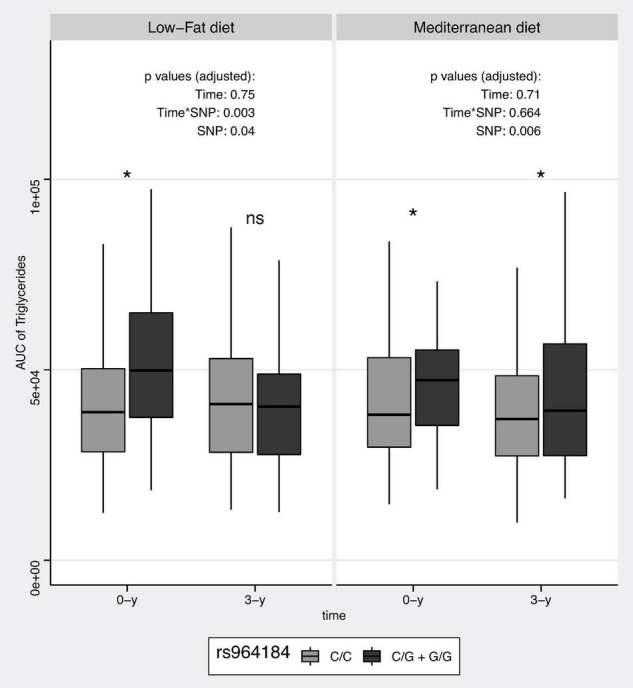
Postprandial AUC of triglycerides according to intervention diet and rs964184 genotypes. Values are shown as box plots, with the median, the approximate quartiles, and the lowest and highest data points. *P*-values were adjusted for age, gender, lipid-lowering drugs, and body mass index. *, *p*-value between genotypes < 0.05; ns, *p*-value between genotypes > 0.05.

Changes in postprandial TG AUC according to LF diet and genotypes are shown in Figure 3. Carriers of the G allele randomized to the LF diet had a higher magnitude of postprandial TG AUC of TG at baseline compared to C/C patients (*p*-value = 0.001). No differences were observed in the AUC of TG between genotypes after 3 years of intervention (*p*-value = 0.744). The *p*-value for the interaction time × SNP was 0.003.

Changes in postprandial TG iAUC by genotypes are shown in Supplementary Figure 2, with a *p*-value for interaction time × SNP = 0.02.

### Mediterranean Diet Intervention

Changes in postprandial TG AUC according to MedDiet and genotypes are shown in Figure 3. At baseline, carriers of the G allele had higher AUC of TG compared to C/C patients (*p*-value = 0.017). After 3 years of Med-diet intervention, this difference remained (*p*-value = 0.04). The *p*-value for the interaction time × SNP was 0.664.

Changes in postprandial TG iAUC by genotypes are shown in Supplementary Figure 2, with a *p*-value for interaction time × SNP = 0.692.

## Discussion

In this study, we investigated the effects of a long-term dietary intervention in CAD patients on postprandial triglyceridemia according to the presence or absence of the G allele at the ZPR1 rs964184 SNP. Our results show that the potential TG-rising effect of the G allele can be differentially modulated by diet. Specifically, carriers of the rs964184 allele risk (G/G or C/G), who, on average, had higher TG levels at baseline than CC carriers, decreased their TGs to levels similar to the CC carriers when following an LF diet for 3 years. Conversely, those G-allele carriers who followed a MedDiet intervention maintained their significantly higher TGs during the duration of the follow-up.

Dietetic interventions that encourage high-quality dietary patterns with moderate caloric intake have been linked to improved cardiovascular risk factor control (44). In that way, the MedDiet has been proposed as a compelling option for the prevention and treatment of cardiovascular disease (41), as well as an alternative to other dietary patterns, such as LF diets (22, 39). Thus, in a subgroup of patients with type 2 diabetes mellitus from the CORDIOPREV trial, we previously assessed the favorable effects of the MedDiet compared to the LF diet on PPL, finding that long-term consumption of the MedDiet improves PPL response compared to consumption of a low-fat diet (37). Furthermore, we reported a gene-diet interaction between a variation in the APOE gene (rs439401) and MedDiet, with T-allele carriers in the MedDiet group showing a more significant decrease in postprandial TG compared with CC subject (38).

Given that increased TG levels, both fasting and non-fasting, have been related to higher CVD risk (45, 46), the results of the current study provide evidence that specific nutritional advice can benefit patients who are genetically predisposed to higher fasting and postprandial TGs and supports the notion of Precision Nutrition as the path to promote better health (47).

Previous studies have reported that polymorphisms in or near the ZPR1-APOA5-A4-C3-A1 gene complex are associated with plasma lipids and cardiovascular risk (33, 35, 48, 49). The results of the current study support previous findings, showing that rs964184 within the ZPR1 locus is associated with fasting plasma TG levels and HDL-c. This relation with fasting TG has been well documented (26, 50), and has been replicated recently in a longitudinal multi-ancestry cohort (European, African, and Asian ancestry) (49). In accordance with our results, in a cohort of 734 overweight or obese participants from the Pounds Lost Trial, the carriers of the G allele exhibited higher fasting TG and lower HDL-c values at baseline. Moreover, in that population, and after a 2-year intervention, a significant interaction between the rs964184 SNP and dietary fat intake (low vs. high) was reported in TC, LDL-c, and HDL-c variations (36). Specifically, G carriers benefited from a low-fat diet more than CC carriers in relation to TC and LDL-c changes. However, in our study, no gene-diet interactions were identified in fasting lipid changes after the 3-year intervention, and the significant differences observed between genotypes in HDL-c and TG values at baseline remained unchanged at the end of both interventions. Additionally, we have investigated this reported interaction more deeply by examining the effects of long-term healthy dietary interventions on postprandial TG metabolism. Dietary fat intake in our study ranged from a minimum of 35% of the total calories as fat (22% MUFA) in the MedDiet group to less than 30% of total calories as fat in the LF diet group (39, 40). Our data demonstrate that, in this patient population, G-allele carriers benefit more from an LF diet than from a MedDiet concerning postprandial TG metabolism. As far as we know, no other studies have evaluated the effect of these diets on rs964184 to modulate the postprandial TG response.

In addition to postprandial metabolism and plasma lipid concentration, different clinical phenomena have been related to the genetic variant studied in the present study. Metabolic syndrome, T2DM, non-alcoholic fatty liver disease (NAFLD), and cardiovascular disease have all been associated with the ZPR1 variation rs964184 among different populations (31, 35, 51). Thus, in a prospective cohort of 185 Spanish patients with primary hypertriglyceridemia, a significant recessive model of risk of NAFLD was reported, with an Odds Ratio of 4.99 in G/G patients vs. C/G + C/C patients (31). Regarding cardiovascular risk, in a study of 725 Caucasian patients with genetically confirmed familial hypercholesterolemia, Paquette et al. (35) found that rs964184 was significantly associated with incident myocardial infarction even after controlling for traditional cardiovascular risk factors. Moreover, in a cohort of 3757 Japanese individuals who either visited outpatient clinics or were admitted to 6 selected hospitals between 2002 and 2012, the G allele of rs964184 SNP was significantly associated with type 2 diabetes prevalence (OR = 1.25) (29), indicating that this SNP may represent a risk factor for all these conditions. In our study, no genotype differences were observed for the risk of diabetes or obesity, as shown in Table 1, probably related to the particular characteristics of this cohort of secondary prevention patients, with a higher prevalence of cardiometabolic abnormalities compared to the general population.

On the other hand, other environmental factors have also been suggested to modulate this genetic variant. Thus, longer sleep duration has been related to the adverse effects of a genetic risk score constructed in 8648 healthy subjects from the Dongfeng-Tongji cohort with a set of 4 polymorphisms in the APOA4-APOA5-ZPR1-BUD13 gene cluster (rs17119975, rs651821, rs7396835, and rs964184) on 5-year triglyceride changes (52). Furthermore, in addition to the influence of genetic background, exercise frequency, hypertension, and education level have been explored as environmental risk factors that may affect serum lipid profiles and diabetes risk. In this way, Li et al. explored in a cohort of 2323 Chinese Han subjects from the Zhejiang Province which genes and environmental factors were associated with type 2 diabetes mellitus risk (30). They reported a set of SNPs and environmental factors that were associated with a higher risk of T2DM, which included rs96418, together with a history of hypertension, regular intake of meat, and waist circumference.

The mechanisms responsible for the reported interaction have not been elucidated. rs964184 is located within the 3-UTR of the ZPR1 gene, which codified a key regulatory protein required for appropriate nucleolar activity in cell proliferation and signal transduction (51). ZPR1’s promoter region has the ability to bind to peroxisome proliferator-activated receptor gamma (PPARG) proteins 1 and 2, which may activate genes involved in glucose and cholesterol metabolism *via* hepatocyte nuclear factor 4 alpha activation (51). On the other hand, a micro-RNA-related mechanism may also be involved as it has been shown for other TG-related gene-diet interactions. MicroRNAs (miRs) are short nucleotide non-coding RNAs that operate as post-transcriptional inhibitors of gene expression by binding to miR recognition sites within their target mRNAs’ 3-UTRs (53). Previous research has shown that SNPs can cause allele-specific regulation by disrupting crucial areas for binding miRs, known as seed sites, as in the case of the lipoprotein lipase (LPL) variant rs13702 *via* interfering with the miR-410 seed site (54, 55). Future studies are needed to discover the possible underlying mechanisms responsible for the described interaction between the rs964184 SNP and PPL.

Our study has some limitations. Given the characteristics of the CORDIOPREV study population, most patients were exposed to the effect of lipid-lowering drugs prescribed for their existing CVD. Although no differences were observed in the baseline use of statins or fibrates, their use has been related to changes in postprandial metabolism (56, 57) that might influence our results. Moreover, a previous study has reported a gender effect related to this SNP (58); however, given the small number of women in our patient population, we could not test this gene-diet-sex interaction. Thus, the generalization of our results should be made with caution and would require validation on external populations. Nevertheless, our study has strengths such as the characterization of the modulation of genetic induced effects of coronary patients who have received a long-term nutritional intervention, as well as the performance of dynamic postprandial studies beyond the determination of fasting parameters. Future studies should evaluate whether this gene-diet interaction may be associated with a decrease in the rate of incident vascular events.

In summary, the development of specific nutritional advice based on genetic differences between individuals on their response to specific dietary patterns may promote better health in patients with cardiometabolic risk factors. Postprandial events related to TG metabolism are important factors related to the development and progression of atherosclerotic disease, and the rs964184 variant in the ZRP1 gene has been identified through GWAS studies as one of the most strongly associated with the magnitude of postprandial TG response. In our study, we describe a novel gene-diet interaction between the long-term consumption of two healthy dietary patterns (LF diet and MedDiet) and the ZPR1 rs964184 SNP on postprandial TG in coronary patients. Therefore, patients with the G-allele at this SNP would benefit more from a low-fat diet to reduce their genetically induced postprandial hyperlipemia. Further studies should confirm whether such modulation would lead to a reduction in the risk of developing cardiovascular disease and investigate the underlying mechanisms that explain this interaction.

## Data Availability Statement

The original contributions presented in this study are publicly available. This data can be found here: Dryad, Dataset, https://datadryad.org/stash/dataset/doi:10.5061/dryad.9cnp5hqmh.

## Ethics Statement

The studies involving human participants were reviewed and approved by the Ethics Committee of Reina Sofía University Hospital (trial protocol 1496/27/03/2009). The patients/participants provided their written informed consent to participate in this study.

## Author Contributions

JL-M and JD-L contributed to the study concept. JL-M, JD-L, and JO critically reviewed the manuscript. JA-D and AA-dL contributed to the design of the manuscript, figures preparation, edition, and manuscript drafting. JA-D, AA-dL, JT-P, FR-C, OR-Z, EY-S, FG-M, MC, RL, and PP-M contributed to the acquisition and analysis of data. All authors gave final approval for all aspects of the work, agreed to be fully accountable for ensuring the integrity and accuracy of the work, and read and approved the final manuscript.

## Conflict of Interest

The authors declare that the research was conducted in the absence of any commercial or financial relationships that could be construed as a potential conflict of interest.

## Publisher’s Note

All claims expressed in this article are solely those of the authors and do not necessarily represent those of their affiliated organizations, or those of the publisher, the editors and the reviewers. Any product that may be evaluated in this article, or claim that may be made by its manufacturer, is not guaranteed or endorsed by the publisher.

## Abbreviations

ApoA1: apolipoprotein A1
ApoB: apolipoprotrein B
AUC: total area under the curve
CAD: coronary artery disease
CVD: cardiovascular disease
CHD: coronary heart disease
CHO: carbohydrates
GWAS: genome-wide association study
HDL-c: high-density lipoprotein cholesterol
Hs-CRP: high sensitive C-reactive protein
iAUC: incremental area under the curve
LDL-c: low-density lipoprotein cholesterol
LF: low-fat
Lp(a): lipoprotein(a)
MedDiet: Mediterranean diet
miRs: microRNAs
MUFA: monounsaturated fatty acids
NAFLD: non-alcoholic fatty liver disease
OFTT: oral fat tolerance test
PPL: postprandial lipemia
PUFA: polyunsaturated fatty acids
SFA: saturated fatty acids
SNP: single nucleotide polymorphism
T2DM: type 2 diabetes mellitus
TG: triglycerides
TRLs: triglycerides-rich-lipoproteins.
