# Supplementary material for: A Gene Variation at the ZPR1 Locus (rs964184) Interacts With the Type of Diet to Modulate Postprandial Triglycerides in Patients With Coronary Artery Disease: From the Coronary Diet Intervention With Olive Oil and Cardiovascular Prevention Study
Source: Front Nutr. 2022 Jun 17;9:885256. doi: 10.3389/fnut.2022.885256 (PMC9247506; doi:10.3389/fnut.2022.885256)
Supplement: Supplementary file 1 [file Data_Sheet_1.docx]

Supplementary Material

**Table S1**. Inclusion and exclusion criteria for CORDIOPREV study.

| Inclusion criteria | 1. Informed consent: All participants will agree to being included in the study by signing the protocol approved by the Reina Sofia University Hospital Clinical Research Ethics Committee 2. Diagnostic criteria: The patients were selected with acute coronary syndrome (unstable angina, acute myocardial infarction) and high-risk chronic CHD according to the following criteria: 3. Acute myocardial infarction: The existence of at least 2 of the following 3 signs: angina-type chest pain (or anginal equivalents), typical ECG changes (appearance of new Q waves and/or changes in ST segments and/or T waves), and a rise in myocardial enzymes (creatine phosphokinase and/or creatine phosphokinase/myoglobin more than twice the normal laboratory limits). The myoglobin value criterion will prevail in case of discrepancies over the total creatine phosphokinase. 4. Unstable angina: Admission to hospital for angina-type chest pains lasting at least 15 min, both at rest and after exercise, which have increased in frequency and duration in recent days or weeks. The latest episode must have occurred at least 48 h before admission and must be accompanied by at least 1 of the following electrocardiographic or analytical changes:   - ST depression of at least 0.5 mm in 2 contiguous leads.  - ST elevation of at least 1 mm in 2 contiguous leads.  - T-wave inversion of at least 2 mm in 2 contiguous leads.  - Positive troponin result.   1. Chronic high-risk ischemic heart disease: patients will be included who have been hospitalized for a coronary event and/or stable angina at least once in the past 2 years and who have undergone diagnostic coronary angiography with evidence of severe coronary disease, which is defined as the existence of an epicardial vessel greater than 2.5 mm in diameter with stenosis of >50%. |
| --- | --- |
| Exclusion criteria | 1. Patients < 20 y of age or >75 y old, or with a life expectancy < 5 years 2. Severe heart failure, NYHA functional class III or IV, with the exception of self-limited episodes of acute heart failure at the time of the acute ischemic event. 3. Severe left ventricular systolic dysfunction (with ejection fraction ≤35%). 4. Patients with restricted capacity to follow the protocol: those unable to follow the prescribed diet for whatever reason, due to personal or family circumstances. 5. Risk factors which are severe or difficult to control: Patients with hypertension and diabetes, where there is organ involvement that limits their survival, were excluded (chronic renal failure with creatinine which is persistently >2.5 mg/dL) and disabling clinical manifestations of cerebral atherosclerosis. 6. Chronic diseases unrelated to coronary risk: severe psychiatric illnesses, chronic conditions requiring treatment that could limit the dietary intervention (chronic renal failure, chronic liver disease, neoplasia under treatment, chronic obstructive pulmonary disease involving respiratory pulmonary failure with home oxygen therapy, endocrine diseases susceptible to decompensation, and diseases of the digestive tract that involve episodes of diarrhea). 7. Participants in other studies: Patients taking part in other studies, at the time of selection or up to 30 d before the study begins, were excluded. |

**Table S2**. Baseline characteristics according to dietary patterns (n=523).

|  | All patients  (n=523) | LF group  (n=255) | MedDiet group  (n=268) | p-value |
| --- | --- | --- | --- | --- |
| Male/Female n | 445/78 | 216/39 | 229/39 | 0.902 |
| Age (years) | 59 ± 0.4 | 59 ± 0.5 | 59 ± 0.6 | 0.755 |
| T2DM n(%) | 230 (44) | 122 (48) | 108 (40) | 0.094 |
| Weight (kg) | 84.6 ± 0.6 | 85.1 ± 0.9 | 84.2 ± 0.9 | 0.448 |
| WC (cm) | 104.1 ± 0.5 | 104.3 ± 0.7 | 103.8 ± 0.7 | 0.602 |
| BMI (kg/m2) | 30.8 ± 0.2 | 30.9 ± 0.3 | 30.7 ± 0.3 | 0.479 |
| TC (mg/dl) | 160 ± 1.4 | 159 ± 1.9 | 161 ± 1.9 | 0.389 |
| LDL-c (mg/dl) | 89 ± 1.1 | 88 ± 1.5 | 91 ± 1.6 | 0.282 |
| HDL-c (mg/dl) | 42 ± 0.4 | 42 ± 0.6 | 43 ± 0.6 | 0.349 |
| TG (mg/dl) | 133 ± 3.0 | 137 ± 4.4 | 130 ± 4.1 | 0.256 |
| ApoA1 (mg/dl) | 130 ± 0.9 | 129 ± 1.2 | 130 ± 1.4 | 0.337 |
| ApoB (mg/dl) | 73 ± 0.8 | 73 ± 1.1 | 74 ± 1.1 | 0.440 |
| Lp(a) (mg/dl) | 40 ±1.8 | 41 ± 2.6 | 39 ± 2.7 | 0.680 |
| Statins n(%) | 457 (87.4) | 231(90.6) | 226 (84.3) | 0.04 |
| Fibrates n(%) | 8 (1.5) | 2 (0.1) | 6(2.2) | 0.287 |

Values are expressed as frequencies (%) or mean ± SEM. Continuous variables have been calculated using the ANOVA test. Qualitative variables were compared using Fisher´s exact test. Abbreviations: ApoA1, apolipoprotein A1, ApoB, apolipoprotein B; BMI, body mass index; cm; centimetres; HDL‐c, high‐density lipoprotein cholesterol; hs‐CRP, high sensitivity C‐reactive protein; kg, kilograms; kg/m2, kilograms per square metre; LDL‐c, low‐density lipoprotein cholesterol; LF, Low fat; Lp(a), lipoprotein a; mg/dL, milligrams per decilitre; MedDiet, Mediterranean diet; mg/L, milligrams per litre; T2DM, type 2 diabetes mellitus; TC, total cholesterol; TG, triglycerides; WC, waist circumference. *P < 0.05.

**Table S3.** Differences (3 years – baseline) in area under the curve and incremental area under the curve of postprandial triglycerides by adjusted univariate analysis

|  | **LF diet** | | **MedDiet** | | **p-value SNP*diet** |
| --- | --- | --- | --- | --- | --- |
|  | C/C (n=188) | C/G+G/G (n=67) | C/C (n=210) | C/G+G/G (n=58) |  |
| **∆_3y-0y_ AUC TG** | -25.8±26350.9 | -13068.4±29471.1 | -2865.3±19584.8 | -4659.3±39430.3 | 0.006 |
| **∆_3y-0y_ iAUC TG** | -148.7±14540.5 | -5388.4±14720.1 | -1765.8±9413.7 | -2801-4±13947.5 | 0.031 |

Values expressed as mean ± SD. P-values for the interaction term SNP*diet were calculated using the ANOVA test adjusted by age, gender, BMI, statins, and fibrates. Abbreviations: AUC, area under the curve; iAUC, incremental area under the curve; LF, low-fat; MedDiet, Mediterranean diet; SNP, single nucleotide polymorphism; TG, triglycerides.

**Figure S1.** Panel A: Changes in adherence to the Mediterranean diet during 3 years of intervention. Values are expressed as mean±SEM. * p<0.001 for comparisons between intervention groups (Low-fat diet vs MedDiet) at each visit. # p<0.001 from baseline in each group of diet. MedDiet, Mediterranean diet group; Low-Fat Diet, low-fat diet group. Panel B: Changes in adherence to the low-fat diet during 3 years of intervention. Values are expressed as mean±SEM. #p<0.001 from baseline in each group of diet. MedDiet, Mediterranean diet group; Low-Fat diet, low-fat diet group.

.

**Figure S2.** Postprandial iAUC of triglycerides according to intervention diet and rs964184 genotypes.

Values are shown as box-plots, with the median, the approximate quartiles, and the lowest and highest data points. P-values were adjusted for age, gender, lipid-lowering drugs, and body mass index.

*, p-value between genotypes <0.05. ns, p-value between genotypes >0.05.
